# Common Mental Disorders in Public Transportation Drivers in Lima, Peru

**DOI:** 10.1371/journal.pone.0101066

**Published:** 2014-06-30

**Authors:** Paulo Ruiz-Grosso, Mariana Ramos, Frine Samalvides, Johann Vega-Dienstmaier, Hever Kruger

**Affiliations:** 1 Mental Health Working Group - Universidad Peruana Cayetano Heredia, Lima, Peru; 2 Alberto Hurtado Medical School – Universidad Peruana Cayetano Heredia, Lima, Peru; 3 Hospital Nacional Cayetano Heredia, Lima, Peru; Asociacion Civil Impacta Salud y Educacion, Peru

## Abstract

**Background:**

Traffic related injuries are leading contributors to burden of disease worldwide. In developing countries a high proportion of them can be attributed to public transportation vehicles. Several mental disorders including alcohol and drug abuse, psychotic disorders, mental stress, productivity pressure, and low monetary income were found predictors of high rates of traffic related injuries in public transportation drivers. The goal of this study was to estimate the prevalence of common mental disorders in the population of public transportation drivers of buses and rickshaws in Lima, Peru.

**Methodology/Principal Findings:**

Cross sectional study. A sample of bus and rickshaw drivers was systematically selected from formal public transportation companies using a snowball approach. Participants completed self-administered questionnaires for assessing major depressive episode, anxiety symptoms, alcohol abuse, and burnout syndrome. Socio demographic information was also collected. The analyses consisted of descriptive measurement of outcomes taking into account both between and within cluster standard deviation (BCSD and WCSD). A total of 278 bus and 227 rickshaw drivers out of 25 companies agreed to participate in the study. BCSD for major depressive episode, anxiety symptoms and burnout syndrome was not found significant (p>0.05). The estimated prevalence of each variable was 13.7% (IC95%: 10.7–16.6%), 24.1% (IC95%: 19.4–28.8%) and 14.1% (IC95%: 10.8–17.4%) respectively. The estimated prevalence of alcohol abuse was 75.4% (IC95%: 69–81.7%, BCSD = 12.2%, WCSD = 41.9%, intra class correlation (ICC): 7.8%).

**Conclusion:**

Common mental disorders such as alcohol abuse, major depressive episode, anxiety symptoms and burnout syndrome presented higher rates in public transportation drivers than general population.

## Introduction

Neuro-psychiatric conditions including mood, anxiety and drug use disorders are known contributors to the global burden of disease [1]. In Peru they contribute with 16%. Alcohol use disorders and unipolar depressive disorders ranked on the top 5 single diagnosis contributing to disability adjusted life years [2]. According to the latest prevalence estimates of mental disorders in Lima, capital city of Peru, the point prevalence of any psychiatric disorders was estimated in 23.5%, 14.6% for any anxiety disorder and 6.7% for depressive disorders. All of those disorders were found to be inversely associated with socio-economic status [3].

Road traffic injuries are one of the top 3 causes of death in people between 5 and 44 years worldwide [4]. The World Health Organization has estimated that fatality rates in low and middle income countries are higher than in high income countries (21.5 vs. 19.5/100, 000); more than 90% of the deaths related to traffic injuries occur in low and middle income countries where victims of road traffic injuries tended to have low income[5], [6].

According to the Burden of Disease Report road traffic injuries are the second contributor to disability in Peru with 13% of the disability adjusted life years, surpassing perinatal conditions, cardiovascular diseases and all infections. Also, they were the first single contributor to burden of disease, above diseases like pneumonia.

It was estimated that 58% of the road traffic injuries were due to car-to-car events, and 27% due to car-to-person events. Approximately 36% of these injuries occurred on Fridays or Saturdays, most of them between 8 am and 2 pm. High speed driving was the main registered cause for road traffic injuries (31%), followed by driver's recklessness (26%) and driving under the influence of alcohol (10%) [7].

Nowadays, Lima's public transportation is being reformulating and redesigned; however, rural trucks (small buses locally known as “combis” and “coasters”) are still the main medium and long-range distance way of transportation. Short distances are covered by the relatively widespread use of rickshaws (locally known as “moto-taxis”), which are more commonly used by population of middle-low and low socio-economic status. These vehicles account for 21.8 and 10% of the reported car accidents in Peru, respectively [7].

This research aims to estimate the prevalence of common mental disorders such as major depressive episode, anxiety symptoms and alcohol use disorders. A precise estimation of the proportion of these conditions among public transportation drivers might provide critical information that may serve to prioritize future strategies for control.

## Methods

### Objectives

This study aimed to estimate the prevalence of common mental disorders in public transportation drivers of both, buses and rickshaws in Lima, Peru. Outcome measures included symptoms compatible with major depressive episode, alcohol abuse, clinically significant anxiety symptoms and burnout syndrome.

### Participants

The study participants were drivers working in buses and rickshaws owned by legally constituted public transportation companies from Carabayllo and San Juan de Miraflores districts (northern and southern districts of Lima respectively). By the time when the study was conducted, each district in Lima was able to provide authorization to transportation companies; however, even when a specific district provided the permission, it did not limit the company's range of operation just to the area under its jurisdiction but allow them to work in whole Lima City. Buses use to cover extensive territories usually involving more than 5 districts. Rickshaw companies tend to be more focalized around specific points of interest such as markets or hospitals; consequently, cover much smaller routes. Legal transportation in Peru is organized in private companies, which are individually authorized to function. Although there is no mandatory employment model, these companies follow one of the two most common modalities of work or a combination of both. In the first one a person or a business society own the vehicles and rent them to individual drivers for a daily rate. In the second, the drivers are the owners of the vehicles a constitute associations with other owners to collectively obtain a permission for a single route. The crew of buses typically consists of two persons: the driver and a collector (locally known as “cobrador”), whose function is to collect passengers' fee. Usually, drivers and collectors meet early in the morning at the company's facilities where are assigned a vehicle and receive authorization to leave at fixed time intervals, according to arriving order.

Data collection took place from January to June of 2010. Given the absence of preliminary data, sample size was initially calculated assuming that the prevalence of individual outcomes on the study population was 50% to maximize the required sample size, with a precision of 5%, design effect of 1.5 due to the snowball sampling, and 10% penalization for each additional outcome measured. The calculated sample size was of 888 individuals. When 25% of the sample was enrolled, we conducted a preliminary analysis of outcomes prevalence to adjust the sample size. The resulting sample size using the same parameters was 406 individuals.

### Study Design

A cross sectional design was used to estimate the prevalence of alcohol abuse, major depressive episode, anxiety symptoms and burnout syndrome. Participants were systematically selected following a snowball sampling technique. Drivers completed a survey that included validated Spanish versions of the Center for Epidemiological Studies Depression Scale (CES-D), the CAGE questionnaire for alcoholism screening, the Zung Self Rating Anxiety Scale (ZSRA) and the Maslach Burnout Inventory (MBI). Additionally, socio-demographic data such as date of birth, sex, marital status, maximal level of instruction reached, religion, number of children, place of birth, years living in Lima, number of working hours during day and night, number of current simultaneous jobs, number of years as public transportation driver, number of infraction tickets on the previous 30 days, 6 months, 5 years and lifetime, monthly individual and family income, and type of health insurance.

### Data Collection

The research team contacted the manager of any single transportation company in both Carabayllo and San Juan de Miraflores, explained the purpose of the study as well as the methods to be used and requested permission for data collection during a company meeting or in a regular day while drivers are waiting for departure authorization. In both occasions, a collective explanation of the study aims, financing and methods were given to all individuals before provide them with the informed consent form. Concerns were privately solved. Individuals who agreed to participate in the study received a copy of the survey. The study personnel remained available at the facilities to solve individual or collective concerns during the questionnaire solving until every individual completed the survey but were previously trained to not interfere or induce any answer. When the process was completed, the manager was asked to voluntarily refer the research team to other companies of the same district for continuing the study. The research team contacted these new companies and repeated the process described before until the sample size was completed.

### Instruments for outcome measurements

#### Center for Epidemiological Studies Depression Scale (CES-D)

The CES-D is a 20-item scale designed to be a screening instrument for depressive disorders in general population. It is supported as a good psychometric test in contexts with high cultural variation such as Latin America [8], [9]. In the present study, a short 5-item version validated in Spanish language was used. This version has showed good discriminative power (sensitivity = 0.96, specificity = 0.93) for a cut-off score ≥6 as well as good internal consistency (Cronbach's alpha = 0.90). This version consisted of 5 items, each one scored from “0” to “3” according to the amount of days on the previous week that the person felt according to the item's premise. The total score varies from 0 to 15. Depressive symptoms suggesting major depressive episode major depressive episode were defined as a score equal as or higher than 6.

#### CAGE Questionnaire

The CAGE questionnaire, named as an acronym form for its items (Cutting down, Annoyance by criticism, Guilty feeling and Eye-openers), has proved to be an effective diagnostic help for alcoholism [10]. A final score of 1 or more predicts high alcoholism probability. This cut-off score showed 92% sensitivity and 74% specificity for a diagnosis of alcoholism defined as alcohol abuse or dependence in Latino population [11].

#### Zung Self Rating Anxiety Scale (ZSAS)

The ZSAS is a self-rated instrument designed to screen for clinically important anxiety symptoms [12]. The ZSAS consists of 20 items, each individually scored from “1” to “4”, making the score vary from 20 to 80. This is usually pondered to a range from 25 to 100, being a score of 50 or higher suggestive of clinically significant anxiety symptoms. A Spanish version validated by experts was used in this study.

#### Maslach Burnout Inventory (MBI)

The MBI is a psychometric instrument designed to measure burnout symptoms grouped in three dimensions: emotional exhaustion, reduced sense of personal accomplishment and depersonalization [13]; a person is considered to present burnout syndrome if their score of at least 2 of the three dimensions is higher than the 75 percentile.

### Statistical Methods

Prevalence was estimated using the random effects univariate linear model to control for effects of clustering by public transportation company. The null hypotheses that the slope representing the prevalence for each variable does not vary across clusters (transport companies) was tested using a chi2 test. Between and within cluster standard deviations is reported if the aforementioned hypothesis was rejected. Stata 11 was used to make the estimations.

### Ethics Statement

The study protocol was reviewed and approved by the Institutional Review Board of the Universidad Peruana Cayetano Heredia. All participants provided written informed consent before completing the self-administered questionnaire.

## Results

Five hundred and five individuals from 25 public transportation companies were recruited. Of them, 278 were bus drivers and 227 were rickshaw drivers.

### Prevalence of Mental Disorders

Overall prevalence of alcoholism was 74.3%; major depressive episode, 13.7%; clinically significant anxiety symptoms, 24.2%; and burnout syndrome, 14.1%. Within cluster variance was found significant (p<0.001) for alcoholism; however, when stratification in bus and rickshaw drivers groups was done, within cluster variance was observed only in bus drivers. Also after stratification, clinically significant anxiety symptoms showed significant within cluster variation (WCV) for the bus drivers group. No statistically significant differences were found on the prevalence of any of the studied mental disorders between bus and rickshaw drivers. Results are summarized on Table 1.

**Table 1:** Prevalence of mental disorders.

		Overall	Bus Drivers	Rickshaw Drivers	p-value**
		n = 505	n = 278	n = 227	
**Alcohol Abuse**	**Prevalence**	**73.2%**	**67.7%**	**79.6%**	
	O-SD	44.3%	46.9%	40.4%	0.090
	BC-SD	17.3%	19.9%	13.3%	
	WC-SD	41.6%	43.7%	39.1%	
	p-value*	<0.001	0.001	0.289	
**Major Depressive Episode**	**Prevalence**	**13.9%**	**15.6%**	**11.9%**	
	O-SD	34.6%	36.3%	32.4%	0.245
	BC-SD	12.4%	14.8%	8.5%	
	WC-SD	33.6%	35.0%	32.1%	
	p-value*	∼1.000	0.230	∼1.000	
**Clinically Significant Anxiety Symptoms**	**Prevalence**	**22.8%**	**23.6%**	**21.9%**	
	O-SD	42.0%	42.6%	41.4%	0.647
	BC-SD	20.3%	25.2%	10.7%	
	WC-SD	40.4%	40.1%	41.0%	
	p-value*	0.092	0.017	∼1.000	
**Burnout Syndrome**	**Prevalence**	**13.9%**	**16.8%**	**11.2%**	
	O-SD	34.7%	37.4%	31.6%	0.131
	BC-SD	15.5%	18.3%	9.5%	
	WC-SD	33.2%	35.6%	30.8%	
	p-value*	∼1.000	0.281	∼1.000	

O-SD: Overall Standard Deviation, BC-SD: Between cluster standard deviation, WC-SD: Within cluster standard deviation.

*p-value for the null hypotheses that between cluster is equal to zero.

**p-value for the null hypotheses that prevalence of measured variable does not vary according to job

### Socio Demographic and Work Related Co-variables

Socio-demographic characteristics of participants are summarized in Table 2. Overall age was 36.4 years, 95.6% of the sample was male, mean number of children was 2.3, 66.4% where married or cohabitants, 7.2% divorced, 64.3% completed secondary school, 19.9% completed superior education, 62.1% were internal migrants and the average time of residence in Lima was of 25.4 years. Results show that when analyzed together, both bus and rickshaw drivers presented significant (p<0.05) WCV in age, number of children, marital status of married or co-habitants, completed high school and graduated studies, and time of residence in Lima. However, when stratifying by job (bus and rickshaw drivers), this pattern changed for number of children, time of residence in Lima, complete secondary school, complete superior education (WCV significant only for bus drivers) and marital status of married or cohabitant (WCV significant only for rickshaw drivers). Statistically significant differences between bus and rickshaw drivers was observed for age (mean = 36.4 vs. 32.3, p<0.001), males (99.3 vs. 92.7%, p<0.001), number of children (mean = 2.3 vs. 2.8, p<0.001), divorced status (9.8 vs. 3.1%) and time of residence in Lima (mean = 28.4 vs. 21.6, p = 0.001).

**Table 2:** Demographic Variables.

		Overall	Bus Drivers	Rickshaw Drivers	p-value**
		n = 505	n = 278	n = 227	
**Age**	**mean**	**36.9**	**40.4**	**32.3**	
	O-SD	12.1	12.1	11.1	<0.001
	BC-SD	6.2	5.9	5.2	
	WC-SD	10.4	10.7	10.2	
	p-value*	<0.001	<0.001	<0.001	
**Male Sex**	**Prevalence**	**96.3%**	**99.3%**	**92.7%**	
	O-SD	20.5%	8.5%	26.1%	<0.001
	BC-SD	6.5%	2.0%	7.2%	
	WC-SD	19.9%	8.3%	25.7%	
	p-value*	0.418	∼1.000	∼1.000	
**Number of children**	**mean**	**2.3**	**2.8**	**1.7**	
	O-SD	1.9	2.0	1.6	<0.001
	BC-SD	0.8	0.7	0.4	
	WC-SD	1.7	1.9	1.6	
	p-value*	<0.001	0.002	0.162	
**Married or Co-habitants**	**Prevalence**	**67.9%**	**71.7%**	**62.5%**	
	O-SD	47.3%	45.1%	48.5%	0.114
	BC-SD	15.0%	15.2%	17.6%	
	WC-SD	45.2%	43.8%	45.4%	
	p-value*	0.002	0.273	0.001	
**Divorced**	**Prevalence**	**6.9%**	**9.8%**	**3.1%**	
	O-SD	25.9%	29.8%	17.4%	0.003
	BC-SD	6.8%	9.9%	5.1%	
	WC-SD	25.2%	28.9%	17.1%	
	p-value*	0.127	0.470	∼1.00	
**Complete Secondary School**	**Prevalence**	**62.6%**	**64.4%**	**61.8%**	
	O-SD	48.0%	48.0%	48.7%	0.671
	BC-SD	15.8%	20.0%	14.0%	
	WC-SD	46.2%	45.5%	47.9%	
	p-value*	0.035	0.015	∼1.000	
**Complete Superior Education**	**Prevalence**	**20.2%**	**22.9%**	**15.9%**	
	O-SD	39.9%	42.1%	36.7%	0.186
	BC-SD	15.5%	17.3%	14.6%	
	WC-SD	37.7%	39.3%	35.2%	
	p-value*	<0.001	<0.001	0.184	
**Migrants**	**Prevalence**	**62.7%**	**63.8%**	**61.3%**	
	O-SD	48.6%	48.1%	48.8%	0.565
	BC-SD	18.2%	15.1%	22.1%	
	WC-SD	47.1%	47.1%	46.9%	
	p-value*	∼1.000	∼1.000	0.354	
**Time of residence in Lima**	**mean**	**25.5**	**28.4**	**21.6**	
	O-SD	14.0	15.5	11.6	0.001
	BC-SD	6.1	6.3	4.4	
	WC-SD	12.9	14.3	11.2	
	p-value*	<0.001	<0.001	0.205	

O-SD: Overall Standard Deviation, BC-SD: Between cluster standard deviation, WC-SD: Within cluster standard deviation.

*p-value for the null hypotheses that between cluster is equal to zero.

**p-value for the null hypotheses that prevalence of measured variable does not vary according to job.

Regarding work-related variables, when bus and rickshaw drivers were analyzed as a single group, mean daytime daily working hours were 12.5 hours while nighttime working hours per week were 21.9. Mean time as public transportation driver was 11 years, number of jobs related to public transportation was 1.4, the number of tickets due to traffic infractions for the last 30 days, last 6 months, last 5 years and lifetime was 0.3, 0.7, 3.1 and 5.4 respectively. Mean personal income was of $316.8 while for family income was $465.2. Taken together, bus and rickshaw drivers showed significant (p<0.05) WCV for every work-related variable, with the exception of monthly family income. When analyzed separately, daytime daily working hours, number of lifetime traffic tickets, monthly personal income and having any kind of health insurance remained with significant (p<0.05) WCV for the bus drivers group but not for the rickshaw drivers one. Time of nighttime work per week, number of traffic tickets on the last 30 days and 6 months presented significant WCV for rickshaw but not for bus drivers. Time being a public transportation driver, number of jobs related to public transportation and number of traffic tickets showed significant WCV for both bus and rickshaw drivers, while monthly familiar income did not show significant WCV for neither group. Significant differences between bus and rickshaw drivers was observed for time of daytime work per day (mean = 13.9 vs. 10.4, p<0.001), number of jobs related to public transportation (mean = 1.6 vs.1.2, p<0.001), monthly personal income (mean = S/. 1 011.2 vs.741.9, p = 0.016), number of tickets for traffic infractions in the last 30 days (mean = 0.7 vs. 0.1, p = 0.023), 6 months (mean = 1.4 vs. 0.1, p<0.001), 5 years (mean = 7 vs. 0.3, p<0.001) and lifetime (mean = 13 vs. 0.5, p<0.001). Results are summarized in Table 3.

**Table 3:** Work related variables.

		Overall	Bus Drivers	Rickshaw Drivers	p-value**
		n = 505	n = 278	n = 227	
	**mean**	**12.3**	**13.9**	**10.4**	
**Time of daytime work per day**	O-SD	4.0	3.3	3.9	<0.001
	BC-SD	2.5	1.8	1.4	
	WC-SD	3.4	3.1	3.7	
	p-value*	<0.001	0.046	0.116	
	**mean**	**21.6**	**24.8**	**18.8**	
**Time of nightime work per week**	O-SD	25.4	28.9	21.5	0.220
	BC-SD	17.4	17.3	17.6	
	WC-SD	22.9	26.7	19.1	
	p-value*	<0.001	0.060	<0.001	
**Time of public transportation driver**	**mean**	**11.2**	**13.2**	**9.0**	
	O-SD	43.7	10.4	62.6	0.678
	BC-SD	35.0	5.8	53.5	
	WC-SD	39.3	9.6	56.3	
	p-value*	<0.001	<0.001	0.003	
**Number of public transportation companies**	**mean**	**1.4**	**1.6**	**1.2**	
	O-SD	1.0	1.3	0.6	<0.001
	BC-SD	0.3	0.3	0.2	
	WC-SD	1.0	1.3	0.6	
	p-value*	0.001	∼1.000	0.245	
**Number of Traffic Tickets on last 30 days**	**mean**	**0.3**	**0.7**	**0.1**	
	O-SD	0.7	0.8	0.6	0.023
	BC-SD	0.5	0.5	0.6	
	WC-SD	0.6	0.7	0.5	
	p-value*	<0.001	0.050	0.002	
**Number of Traffic Tickets on last 6 months**	**mean**	**0.7**	**1.4**	**0.1**	
	O-SD	1.2	1.5	0.3	<0.001
	BC-SD	0.7	0.6	0.2	
	WC-SD	1.0	1.4	0.3	
	p-value*	<0.001	0.413	0.009	
**Number of Traffic Tickets on last 5 years**	**mean**	**3.3**	**7.0**	**0.3**	
	O-SD	6.3	8.0	0.6	<0.001
	BC-SD	7.2	8.3	0.5	
	WC-SD	4.4	6.6	0.6	
	p-value*	<0.001	<0.001	0.001	
**Number of lifetime traffic tickets**	**mean**	**5.4**	**13.0**	**0.5**	
	O-SD	12.9	17.1	1.8	<0.001
	BC-SD	10.5	11.6	0.7	
	WC-SD	9.4	14.2	1.7	
	p-value*	<0.001	<0.001	0.050	
**Monthly personal income**	**mean**	**887.5**	**1011.2**	**741.9**	
	O-SD	905.8	772.1	1024.4	0.016
	BC-SD	374.9	431.0	207.6	
	WC-SD	850.4	695.0	1005.0	
	p-value*	<0.001	<0.001	∼1.000	
**Monthly familiar income**	**mean**	**1299.7**	**1300.9**	**1271.4**	
	O-SD	1203.6	791.4	1597.8	0.872
	BC-SD	524.4	422.1	699.0	
	WC-SD	1151.7	736.6	1537.4	
	p-value*	∼1.000	∼1.000	∼1.000	
**Have health insurance**	**prevalence**	**36.1%**	**36.8%**	**35.3%**	
	O-SD	48.1%	48.3%	47.9%	0.637
	BC-SD	21.6%	26.8%	11.5%	
	WC-SD	45.6%	44.6%	46.9%	
	p-value*	<0.001	0.001	∼1.000	

O-SD: Overall Standard Deviation, BC-SD: Between cluster standard deviation, WC-SD: Within cluster standard deviation.

*p-value for the null hypotheses that between cluster is equal to zero.

**p-value for the null hypotheses that prevalence of measured variable does not vary according to job.

## Discussion

Before discussing the significance of findings presented, some limitations of the study must be discussed. According to Lima's Directorate of Urban Transportation disclosed data, 42 000 vehicles used for public transportation currently exists. This includes both legal and illegal companies, and even when the proportion of informal companies is not currently know, it is evident that it consists of a considerable number of units. This is important for interpretation of results, as we only present data corresponding to legal companies. The impact of the omission of informal driver's data is difficult to predict. Drivers working for illegal companies might present poorer mental health results due to lower work standards, excessive workload and the intrinsic stress of working in an illegal work. Also, it noteworthy that sample size was calculated in order to be able to estimate the prevalence of mental disorders, and the separation between bus and rickshaw drivers presented in this research should be interpreted as the result of exploratory analysis, and the results taken with caution when then null hypotheses of the test was not rejected, as this might be due to insufficient statistical power.

The results show that except that for alcohol abuse, common mental disorders were uniformly distributed among the studied clusters of public transportation companies. The estimated point prevalence of major depressive episode (13.7%) was found to be higher than what is reported in the latest population based estimation for Lima City (∼6%). It was higher than what was measured on the same study for the male subpopulation (3%) and for socio-economic status in which basic needs (food and dress) were fulfilled (8.6%) [3]. However, the prevalence of major depressive episode was similar to what was found in truck drivers in Brazil (13.6%) [14].

Point estimates of the prevalence of anxiety symptoms (24.1%) were also higher in the sample population than what is reported for homologues estimates on Lima's general population (14.6%), male general population (10.7%) or even on socio-economic strata where not even basic needs are fulfilled (16.2%).

Prevalence of alcohol abuse (75.4%) was also found to be higher than what is reported for Lima's general population (21.8%) [3]. To make a proper interpretation on the prevalence estimates for alcohol abuse, we need to take into account that the BCSD was found significant (12.2%), which means that the prevalence of alcohol abuse varies across public transportation companies. This being said, research of alcohol intake at various workplace scenarios might help understand these findings. A study conducted on a sample of Brazilian garbage collectors by *dos Santos Mambuchi et al* reported that approximately 15% of the male workers that consumed alcohol presented criteria for alcohol dependence [15]; in India, *Chagas et al* found the frequency of hazardous drinkers to be of 21.3%[16]; and Hermansson et al, reported that 23% of Swedish workers were positive for alcohol use disorders [17]. All the estimates show that the particular population of public transportation drivers in Lima presents a higher prevalence of alcohol abuse than what is reported for workers in other developing countries; however, other studies of alcohol use among drivers tend to focus on driving under its influence and are not suitable for comparison with the present results.

In order to understand the higher prevalence estimates found on this research, complementary socio-demographic and work related data presented in Tables 2 and 3 might be crucial.

Higher rates of alcoholism could be explained by the preponderance of males (95%) on the sample, as male gender has been considered as risk factor, even if the gap between genders seems to be closing [18]–[20]. Also, the relatively low specificity of the CAGE could account for over estimation of the prevalence.

Around 60% of both rickshaw and bus drivers were within-country migrants, which has been related to a higher probability of presenting depressive symptoms and was also suggested that might present a higher intake of alcohol, due to stress generated by migration or as a coping mechanism [21]–[26].

Burnout symptoms consist of the emotional exhaustion, reduced sense of personal accomplishment and depersonalization dimensions as measured by the Maslach Burnout inventory. The results show that roughly 14% of the sample is likely to be suffering with significantly burnout symptoms. Adverse working conditions (Table 3), might contribute to the levels of Burnout and through this mechanism modify the likelihood of presenting affective and substance use disorders [15], [27].

Even if bus and rickshaw drivers were found to be different on key possible predictor variables of common mental disorders such as age, proportion of males, time of daytime work per day, number of jobs and number of traffic tickets and personal income, no statistically significant difference was found across these groups in terms of prevalence of the studied mental disorders.
